# Perceived Emotional Invalidation, Psychological Distress and Relationship Satisfaction in Couples: An Actor–Partner Interdependence Mediation Analysis

**DOI:** 10.1177/00332941241279372

**Published:** 2024-09-02

**Authors:** Tânia Brandão

**Affiliations:** William James Center for Research, 56068 Ispa - Instituto Universitário, Lisboa, Portugal

**Keywords:** perceived Emotional invalidation, psychological distress, couple relationship satisfaction, actor-partner interdependence model

## Abstract

Research has demonstrated a clear link between perceived emotional invalidation and increased psychological distress. However, available studies have predominantly focused on individual data, and leave the impact on relationship satisfaction largely unexplored. Considering the systemic-transactional model, our study aimed to examine the association between perceived emotional invalidation, psychological distress, and couple relationship satisfaction from a dyadic perspective. We conducted a cross-sectional study involving 240 mixed-gender couples from Portugal employing the Actor-Partner Interdependence Mediational Model to analyse the data to examine actor and partner direct and indirect effects. Results showed that, for both women and men, own perceived emotional invalidation was associated with own psychological distress but not with own relationship satisfaction. Also, own psychological distress was associated with own relationship satisfaction but only for women. Finally, one actor and one partner indirect effects were found. Women perceived emotional invalidation was associated with their own psychological distress which in turn contributed to lower levels of relationship satisfaction for both them and their partners. This study contributes to a deeper understanding of the dyadic effects of perceived emotional invalidation on psychological distress and relationship satisfaction within romantic relationships identifying interpersonal emotional dynamics as an important target of interventions.

## Introduction

In the context of romantic relationships, partners play a pivotal role in shaping each other’s shared experiences and perceptions, both in terms of behavioural dynamics and emotional aspects (Butler & Randall, 2013; Mazzuca et al., 2019; Segrin, 2001), especially emotional states between intimate partners co-fluctuate in their daily lives (Butler, 2011). This dynamic interplay underscores the importance of considering both partners’ perspectives and experiences when studying relationship processes. For many years, the study of stress processes primarily revolved around individual focused models that originated outside the field of marriage and family therapy (Falconier et al., 2015). However, recent theoretical advancements provide a more detailed understanding of stress as a dyadic phenomenon (Bodenmann, 1995; Randall & Bodenmann, 2017). These proposals emphasize that daily stressors originating from outside the relationship can infiltrate the relationship, resulting in stress within the relationship - a phenomenon also referred to as *stress spillover* (i.e., spillover of external stress into the dyad) (Bodenmann, 1995). The systemic-transactional model (STM) proposed by Bodenmann (1995, 1997) emphasizes both partners’ interdependence and reciprocal influence in the stress and coping processes.

### Perceived emotion invalidation and relationship satisfaction

In this study, we focus our attention on a specific external stressor - *perceived emotional invalidation*, that is, the experience of having one’s emotions dismissed or misunderstood by others. External stressors, such as perceived emotional invalidation, are likely to directly impact individuals’ relationship satisfaction and indirectly via increasing psychological distress (Bodenmann et al., 2007; Falconier et al., 2015; Ledermann et al., 2010). Many previous studies have primarily concentrated on either assessing the influence of external stressors on individual psychological distress (e.g., Serido et al., 2004) or examining its effects on couples’ functioning (e.g., Neff & Karney, 2009; Totenhagen et al., 2012), but there has been limited research that simultaneously considers both aspects and using a dyadic perspective. However, available evidence indicates that emotions significantly influence partners’ perceptions of their relationship (Bloch et al., 2014; Caughlin & Huston, 2006) and contribute to variations in relationship quality (Brock et al., 2019). As pointed by the interpersonal process model of intimacy (Reis & Shaver, 1988), emotion processes, such as partner responsiveness, are essential in shaping couples’ intimacy. There are no studies examining the effect of perceived emotional invalidation on relationship satisfaction using a dyadic approach. However, studies using the actor-partner interdependence model (APIM; Kenny et al., 2006) and exploring the effect of emotional processes (e.g., emotion regulation) on relationship satisfaction have found that men’s emotional experiences are linked to their personal perceptions of relationship satisfaction; in contrast, women’s emotional experiences not only impact their own relationship satisfaction but also their partner’s satisfaction (Bloch et al., 2014; Rusu et al., 2019; Samios & Khatri, 2019; Xu et al., 2023).

### Perceived emotion invalidation and psychological distress

Emotion regulation theories, including the emotion dysregulation model for mood and anxiety disorders, propose that individual differences in how emotions are experienced and managed significantly influence both positive and negative affect. Ineffective regulation of these emotions can lead to psychological distress (Hofmann et al., 2012). While this model primarily focuses on intrapersonal factors, it is now recognized that social sharing can be an important process in emotion regulation. However, when individuals share/express their emotions with others and are met with responses that invalidate their feelings, it can contribute to psychological distress by making them feel emotionally invalidated (Rimé, 2009; Schreiber & Veilleux, 2022). Perceived emotion invalidation can be defined as a social exchange wherein an individual’s expressed emotions or affective experiences are met with responses that are perceived as implying their emotions are incorrect or inappropriate (Zielinski & Veilleux, 2018).

Recent empirical studies employing an intraindividual approach have established a significant association between perceived emotion invalidation and psychological distress, encompassing symptoms of depression, anxiety, and stress (Brandão et al., 2022; Schreiber & Veilleux, 2022; Zielinski et al., 2022). Indeed, sharing emotions with others has been demonstrated as an effective and beneficial emotion regulation strategy, as it fosters a sense of support and affiliation (Rimé, 2009). Previous studies have underscored the significant role of others’ understanding, encompassing an empathetic grasp of individuals’ emotions and feelings, in promoting overall well-being (Lun et al., 2008). However, the impact of sharing emotions can vary when individuals encounter responses that suggest their emotions are unacceptable or inappropriate. This phenomenon, known as perceived emotion invalidation, can lead to feelings of emotional invalidation (Zielinski & Veilleux, 2018), subsequently contributing to psychological distress. For instance, in an ecological momentary assessment study, perceived emotional invalidation was identified as an emotional vulnerability factor due to its association with reduced positive affect (Zielinski et al., 2022). Thus, the experience of having one’s emotions dismissed or misunderstood by others can profoundly influence an individual’s emotional state, highlighting the critical role of supportive and validating social interactions in mitigating psychological distress.

Few studies have employed a dyadic approach to investigate emotional processes and their impact on couple’s distress. Available research suggests that emotion processes (e.g., co-brooding, i.e., the repetitive and unwanted disclosure of negative content to a partner) are likely to influence own and partner symptoms of adjustment disorder and depression in couples (Horn & Maercker, 2016).

### Psychological distress and relationship satisfaction

Psychological distress constitutes a significant dimension in an individual’s life, encompassing a distressing array of mental and physical symptoms intricately linked to the natural ebbs and flows of mood (VandenBos, 2007). Extensive research has consistently demonstrated that heightened levels of psychological distress can significantly impact various aspects of both intrapersonal and interpersonal realms, affecting psychological well-being, life satisfaction, and family relationships (Henning et al., 2007; Stein & Heimberg, 2004). Although the relationships between psychopathological symptoms are often bidirectional, certain models, such as the interactional model of depression and the stress generation model (e.g., Rehman et al., 2008), emphasize the significant impact of depression on relationship satisfaction.

Specifically related to the role of psychological distress in relationship satisfaction, Randall and Bodenmann (2009) found evidence that everyday stress is often linked to relationship deterioration. This impact can be better understood through dyadic theories of stress. For instance, Bodenmann’s model (1995) sheds light on the effects of chronic daily stress on close relationships. It highlights how various minor processes (e.g., spending less time together, reduced self-disclosure, impaired communication, and increased risk of psychological issues) can cumulatively lead to negative relationship outcomes, including decreased relationship satisfaction.

Also, individuals dealing with psychological distress often exhibit dysfunctional communication patterns characterized by negative behaviours such as criticism, complaints, hostility, as well as poor listening skills (Fincham et al., 2018). This impaired communication can hinder healthy relationships and give rise to conflicts, further exacerbating psychological distress in couples. Also, studies have shown that couples dealing with psychological distress or depressive symptoms usually experience lower levels of relationship satisfaction (Bodenmann et al., 2007; Tolpin et al., 2006). The well-being of individuals can be substantially affected by the actions and situations of those in their close proximity (Reis, 2012). Therefore, it is imperative to investigate the factors that contribute to individual variations in the experience of psychological distress and their implications for relationship satisfaction.

Regarding evidence from dyadic studies, some studies have investigated the role of psychological distress and its impact on relationship satisfaction. For example, in one study, the authors found that relationship satisfaction was influenced by an individual’s own levels of anxiety and depression (i.e., actor effects) and by their spouse’s depression levels (i.e., partner effects), with no gender differences (Whisman et al., 2004). Gana et al. (2016) also found actor and partner effects of negative mood (depression and anxiety) on relationship quality. Specifically, except for men’s anxious mood predicting their own relationship quality, all actor effects were statistically significant; also, two significant partner effects were identified with women’s anxious mood being associated with men’s relationship quality, but their depressive mood was not, and with men’s depressive mood influencing women’s relationship quality but their anxious mood did not. In one study, however, only one actor effect for men was found between depression and relationship satisfaction; actor effect for women and partner effects were not significant (Meyer et al., 2019). It is important to note that this study included only 84 couples.

Also, in one study exploring couples’ sexual satisfaction, a construct strongly linked to relationship satisfaction, it was found that men’s anxiety and stress levels were linked to their own sexual satisfaction. Furthermore, men’s depression was connected to both their own sexual satisfaction and that of their partners. Also, women’s depression and stress influenced their own sexual satisfaction but did not affect their partners’ satisfaction. Women’s anxiety did not correlate with sexual satisfaction for either themselves or their partners (Karakose et al., 2023).

### The present study

This study aimed to investigate the potential link between perceived emotion invalidation, psychological distress - specifically in terms of depression, anxiety, and stress, and couple relationship satisfaction within a sample of mixed-gender couples. Prior research has consistently highlighted perceived emotion invalidation as a significant factor associated with psychological distress in individual contexts (Brandão et al., 2022; Schreiber & Veilleux, 2022; Zielinski et al., 2022) as well as the associations between psychological distress and relationship satisfaction (Bodenmann et al., 2007; Tolpin et al., 2006). However, the crucial dyadic perspective has been overlooked in these investigations. Building on these insights, our study sought to examine the direct effect of perceived emotional invalidation on couple’s relationship satisfaction as well as to examine the indirect effect via psychological distress.

We hypothesized that an individual’s own perceived emotional invalidation would be negatively associated with their own relationship satisfaction (H1), individuals’ own perceived emotional invalidation would be negatively associated with their own psychological distress (H2), and that individuals’ own psychological distress would be negatively associated with their own relationship satisfaction (H3). The same actor associations are expected for men and women.

By incorporating a dyadic approach, our research aimed to provide a comprehensive understanding of how perceived emotion invalidation operates within romantic relationships, impacting the psychological distress and relationship satisfaction of both partners. We not only examined the association between an individual’s own perceived emotional invalidation, psychological distress, and relationship satisfaction, but also delved into the notion of partner effects on this link considering the concept of contagion effects, wherein one partner’s emotional state influences the emotional experience of the other partner (Schoebi & Randall, 2015). Additionally, it aligns with the stress spillover perspective, which suggests that stressors originating from outside the relationship can lead to stress within the relationship for both members of the couple (Bodenmann, 1995).

Thus, we hypothesized that one partner’s perception of emotional invalidation would be negatively associated with the other partner’s relationship satisfaction (H4), one partner’s perception of emotional invalidation would be negatively associated with the other partner’s psychological distress (H5), and that one partner’s psychological distress would be negatively associated with the other partner’s relationship satisfaction (H6). Based on previous studies, we expected different partner associations for men and women. Specifically, we hypothesised that women’s variables (perceived emotional invalidation and psychological distress) would impact their partners’ relationship satisfaction.

Finally, we hypothesized that the links between perception of emotional invalidation and relationship satisfaction would happen via own psychological distress (H7) and partners psychological distress (H8) for both women and men.

## Method

### Participants

This cross-sectional study involved a sample of 240 mixed-gender dyads, totalling 480 individuals. The women’s ages ranged from 19 to 68 years, with a mean age of 38.07 years (*SD* = 13.20). Similarly, the men’s ages ranged from 18 to 67 years, with a mean age of 39.87 years (*SD* = 13.36). All participants were engaged in committed mixed-gender romantic relationships, with an average relationship duration of 12.59 years (*SD* = 11.41).

Regarding education, a significant portion of the sample demonstrated a high level of educational attainment, with 62.3% of women and 71.3% of men reporting at least 12 years of education. Professionally, most women and men were actively employed, comprising 70.3% of women and 80.03% of men. Additionally, 10% of women and 5% of men reported being unemployed, while 16.8% of women and 10.4% of men identified as university students or student-workers.

## Measures

### Perceived emotional invalidation

Perceived emotional invalidation was measured using the Perceived Invalidation of Emotion Scale (PIES; Zielinski & Veilleux, 2018; Portuguese validation: Brandão et al., 2021). PIES is a 10-item self-report instrument that measures perceived emotion invalidation (item example “*When I share how I’m feeling, others don’t seem to understand why I feel the way that I do*”). Items are rated in a Likert-type scale ranging from 1 (*almost never; 0-10%)* to 5 (*almost always; 91-100%)* and averaged to create a mean invalidation score. The alpha coefficient for this sample was .82 for women and .79 for men.

### Psychological distress

Psychological distress was measured with the Depression, Anxiety, and Stress Scale – 21 (DASS-21; Lovibond & Lovibond, 1995; Portuguese Version: Pais-Ribeiro et al., 2004). This self-report scale measures levels of depression, anxiety, and stress using 21 items that are scored in a Likert scale ranging from 0 (*did not apply to me at all*) to 4 (*applied to me very much, or most of the time*). Each dimension is composed of 7 items: depression (item example: “*I couldn’t seem to experience any positive feeling at all*”), anxiety (item example: “*I was aware of dryness of my mouth*”), and stress (item example “*I found it hard to wind down*”). In this study, we averaged the three dimensions to create a single dimension labelled psychological distress, as done previously in Schreiber and Veilleux (2022). The alpha coefficient for this sample was .94 for women and .91 for men for the total score.

### Relationship satisfaction

Perceived relationship satisfaction was measured with a single-item scale (“*How do you rate the quality of your relationship”?*) as done in previous studies (e.g., Moreira et al., 2011). A 6-point Likert scale ranging from 1 (*not satisfied*) to 6 (*totally satisfied*) was used.

### Procedure

The study was approved by a University Ethics Committee and was conducted following the principles of the Helsinki Declaration and later amends. All participants received an information and consent form, and the questionnaires to fill out in paper format at their home. Data was collected by psychology students using a snowball method strategy. Adult (>18 years) living in Portugal involved in a romantic relationship were invited to participate in the study along with their partner. Due to the limited participation of same-gender couples (*n* = 5), they were not included in this study. Participants were instructed to fill out the questionnaires separately. A numeric code was used to match the dyadic data and to guarantee anonymity. Confidentiality was also assured. Participants did not receive any kind of incentives.

### Data analysis

Descriptive and Pearson correlations were performed in SPSS (version 28). Correlation coefficients >0.1 represent a small effect, >0.3 a medium effect and >0.5 a large effect (Field, 2024).

The omnibus chi-square test (Ackermann et al., 2011; Kenny et al., 2006) was used to test whether the data were empirically distinguishable using the APIM_SEM app developed by Stas et al. (2018) that uses the R-package lavaan for fitting structural equation modelling and using the maximum likelihood estimation (Rosseel, 2012). A non-significant chi-square value indicates that the dyadic partner is indistinguishable (Kenny et al., 2006; Stas et al., 2018).

The Actor-Partner Interdependence Model extended to Mediation (APIMeM) (Ledermann et al., 2011) was runed in the SPSS macro MEDYAD developed by Coutts et al. (2019). MEDYAD is a computational macro for SPSS that utilizes regression analysis to perform mediation analysis specifically tailored for distinguishable dyadic data. It calculates actor direct effects (i.e., the impact of an individual’s variable on their own outcome), partner direct effects (i.e., the impact of an individual’s variable on their partner’s outcome), actor indirect effects (i.e., the impact of an individual’s variable on their own outcome through one own intervening variables), and partner indirect effects (i.e., the impact of an individual’s variable on their own or partner’s outcome through one own or partner intervening variables).

MEDYAD employs bootstrap methods to compute indirect effects, which are considered significant when the confidence intervals do not encompass 0. Unstandardized values are reported.

## Results

### Preliminary analyses

Descriptive statistics and correlations among study variables are presented in Table 1. For both women and men, their own perceived invalidation was positively correlated with their own and their partner’s psychological distress (small to moderate correlations). Own and partner perceived emotional invalidation was negatively linked to own and partner relationship satisfaction (small to moderate correlations). For women, their own psychological distress was moderately negatively linked to their own and their partner’s relationship satisfaction, but for men these associations were not significant.

**Table 1.** Descriptive Statistics and Correlations Among Study Variables.

	M (SD)	1	2	3	4	5	6
1. Invalidation women	2.01 (.61)	—					
2. Invalidation men	2.04 (.59)	.248***	—				
3. Psychological distress women	1.61 (.56)	.280***	.185**	—			
4. Psychological distress men	1.46 (.44)	.143*	.319***	.211**	—		
5. Relationship satisfaction women	3.71 (.49)	−.205**	−.202**	−.278***	−.024	—	
6. Relationship satisfaction men	3.76 (.46)	−.279***	−.170*	−.280***	−0.87	.574***	—
7. Age women	38.07 (13.20)	.089	−.094	−.281**	−.225**	−.182**	−.083
8. Age men	39.87 (13.36)	.146*	−.020	−.247**	−.174**	.237**	−.115
9. Relationship length (years)	12.59 (11.41)	.101	.048	−.232**	−.130	−.235**	.014

*Note.* *p < .05; **p < .01; ***p < .001.

Own and partner perceived invalidation as well as own and partner psychological distress were moderately positively correlated. Also, own and partner relationship satisfaction were strongly positively correlated.

Men’s age (i.e., being older) was associated with increased perceived invalidation by women. Both women’s and men’s age (i.e., being older), as well as relationship length (i.e., have longer relationships), were linked to higher psychological distress in women. However, women’s and men’s age (i.e., being younger), as well as relationship length (i.e., have longer relationships), were linked to higher relationship satisfaction in women. Finally, both women’s and men’s age were associated with psychological distress in men.

As participants’ age and relationship length were significantly associated with the study variables, they were included as covariates in the model. However, in the model, only women’s age was significantly associated with men’s psychological distress (*p* < .05). Therefore, to achieve a more parsimonious model, men’s age and relationship length were removed from the model.

### APIM model

#### Test of distinguishability

The omnibus chi-square test of distinguishability showed that the dyadic partner was distinguishable according to the variable gender (female vs. male) (*χ*^*2*^ [6] = 30.58, *p* < .001).

#### Actor, partner, and indirect effects

**Figure 1.**
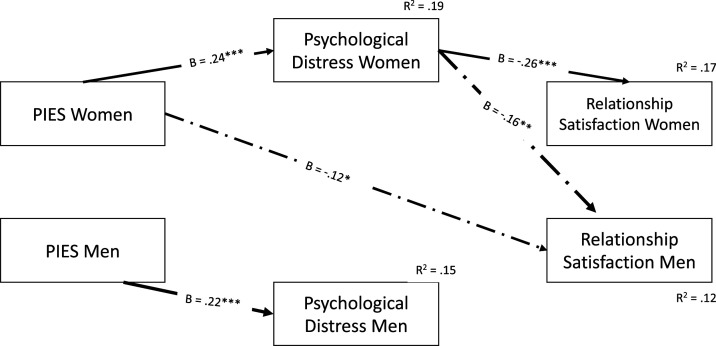
Actor-partner interdependence mediational model (controlling for women’s age). *Note*. Only significant effects are presented. *p < .05; **p < .01; ***p < .001.

Actor and partner effects are displayed in Figure 1 (only significant results are presented) and actor and partner indirect effects are presented in Table 2.

**Table 2.** Actor and Partner Indirect Effects.

Effect	*B*	95%CI	
		Lower	Upper
PIES women - > Psychological distress women - > Relationship satisfaction women	**−.06**	**−.12**	**−.02**
PIES women - > Psychological distress men - > Relationship satisfaction women	.00	−.01	.01
PIES women - > Psychological distress women - > Relationship satisfaction men	**−.04**	**−.10**	**−.00**
PIES women - > Psychological distress men - > Relationship satisfaction women men	−.00	−.01	.01
PIES men - > Psychological distress women - > Relationship satisfaction women	−.02	−.06	.01
PIES men - > Psychological distress men - > Relationship satisfaction women	.00	−.03	.04
PIES men - > Psychological distress women - > Relationship satisfaction men	−.02	−.04	.01
PIES men - > Psychological distress men - > Relationship satisfaction women men	−.00	−.04	.03

*Note.* Controlling for women’s age.

We found two actor effects. Own perceived emotional invalidation was positively associated with own psychological distress for both women and men; and own psychological distress was negatively associated with own relationship satisfaction but only for women. Also, we found two partner effects: own perceived emotional invalidation was negatively associated with partners’ relationship satisfaction but only for women; and own psychological distress was negatively associated with partners’ relationship satisfaction but also only for women.

In terms of indirect effects, we found one actor indirect effect: women perceived emotional invalidation was associated with their levels of psychological distress, which in turn was associated with their own relationship satisfaction. And one partner indirect effect: women perceived emotional invalidation was associated with their levels of psychological distress, which in turn was associated with their partners’ relationship satisfaction. For men, none of the indirect effects were significant.

## Discussion

The aim of this study was to examine the links between perceived emotional invalidation, psychological distress, and couple relationship satisfaction using a dyadic approach. Prior studies have explored the connection between perceived emotional invalidation and psychological distress among individuals (Brandão et al., 2022; Schreiber & Veilleux, 2022; Zielinski et al., 2022). However, there is a significant gap in the literature when it comes to understanding these associations in the context of couples and how external stressors, such as having their emotions seen as rejected or invalidated by others, impact own and partners’ psychological distress and, consequently, couple relationship satisfaction.

To address this research gap, our study takes a closer look at not only the individual effects but also the effects on partners within couples concerning perceived emotional invalidation, psychological distress, and couple relationship satisfaction. We used a dyadic approach to examine these dynamics, aiming to uncover the interdependence and reciprocal influences within romantic partnerships. This comprehensive investigation is essential for a deeper understanding of emotional and relational dynamics in couples and their implications, which can contribute to advancements in interpersonal emotion regulation and couple therapy research.

Our H1 which posited that one’s own perceived emotional invalidation would affect their own relationship satisfaction, was not supported for either women or men. While previous studies have shown that emotional experiences are likely to influence perceptions of relationship satisfaction (e.g., Bloch et al., 2014; Caughlin & Huston, 2006), this is the first study to specifically assess the effects of perceived emotional invalidation. While perceived emotional invalidation encompasses overall perceptions, not just those related to partners’ behaviours, it would be expected that such type of stressor would negatively impact relationship satisfaction (Bodenmann et al., 2007; Falconier et al., 2015; Ledermann et al., 2010). However, our findings did not support this expectation. It is possible that several factors contributed to this result. First, individuals may compartmentalize their experiences of emotional invalidation (Bowins, 2004), separating these from their overall assessment of relationship satisfaction. Also, perceived emotional invalidation may only be detrimental for relationship satisfaction in the presence of other difficulties (e.g., attachment insecurity or lack of social support) or eventually might influence other aspects of individual functioning, including psychological distress, as discussed below, or relationship dynamics such as communication patterns or conflict resolution, rather than overall relationship satisfaction (e.g., Tan et al., 2012). Thus, future studies should further explore these associations. Furthermore, measurement issues might have affected our ability to detect a significant relationship, as the one-item question for assessing relationship satisfaction may not have been sensitive enough to capture the nuances of relationship satisfaction.

Our H2 was empirically supported as we found a significant positive association between an individual’s own perceived emotional invalidation and their own psychological distress, which is consistent with previous research (Brandão et al., 2022; Schreiber & Veilleux, 2022; Zielinski et al., 2022). These findings bolster the notion that perceived emotional invalidation plays a pivotal role in shaping individuals’ psychological functioning. When individuals perceive that their emotions are considered undesirable or unacceptable by others, it appears to heighten their psychological distress. This perception of emotional invalidation may restrict individuals’ expression of emotions, a factor closely linked to feelings of support and affiliation, and subsequently increase the use of expressive suppression, an emotion regulation strategy usually associated with elevated psychological distress as suggested by previous studies (Brandão et al., 2022; Pennebaker, 1997; Rimé, 2009). It is important to note that the perceived emotional invalidation reported is based on generic perceptions, which can include individuals other than romantic partners. While caution is needed, it also seems to suggest that regardless of the person involved, the experience of emotional invalidation is likely to influence an individual’s psychological distress. Interestingly, these associations were equally evident for both women and men, highlighting the gender-invariant nature of this relationship.

Regarding our H3, it was partially confirmed since psychological distress was negatively associated with relationship satisfaction but only for women. It seems that psychological distress appears to influence relationship satisfaction (Bodenmann et al., 2007; Randall & Bodenmann, 2009; Tolpin et al., 2006), more than perceived emotional invalidation. Consistent with previous research, individuals experiencing higher levels of psychological distress may find their relationship satisfaction diminished, likely due to the detrimental impact distress has on couple communication and interaction (Fincham et al., 2018). However, our findings indicate that this was significant only for women. Women are generally more likely to express and process their emotions openly compared to men (Chaplin, 2015). This greater emotional expressiveness resulting from the psychological distress experienced can more readily spill over into their perceptions of relationship satisfaction. Additionally, men often cope with negative mood by distracting themselves or avoiding these feelings, whereas women are more likely to confront their negative mood directly or ruminate on them (Gabriel et al., 2010). This difference in mood regulation can impact women’s relationship satisfaction more significantly than men’s.

Regarding partner effects, our H4 was partially confirmed. Women perceived emotional invalidation was not directly associated with their own relationship satisfaction, but it was negatively directly linked to their partners’ relationship satisfaction. This finding supports the idea that external stressors, like perceiving one’s emotions as being invalidated or criticized by others, can contribute to influence the couple relationship satisfaction, which is consistent with the broader framework of stress spillover outlined in theoretical advancements (Bodenmann, 1995; Randall & Bodenmann, 2017). Also, these results align with previous literature in which women’s emotional experiences impact their partner’s satisfaction (Bloch et al., 2014; Rusu et al., 2019; Samios & Khatri, 2019; Xu et al., 2023). Emotions are often contagious, and when one partner feels invalidated or distressed, it can spillover into the relationship (Schoebi & Randall, 2015). The partner might pick up on these negative emotions, leading to lower satisfaction in the relationship. It is also plausible to hypothesize that when women experience emotional invalidation from others, it may lead to adopting a less constructive approach to regulating emotions or to more difficulties in effectively communicating with their partners. This, in turn, can potentially impact their partner’s satisfaction within the relationship due to difficulties in couple’s communication and interaction (Fincham et al., 2018). Yet, since relationship satisfaction was measured using a single-item question, caution is warranted when interpreting this finding.

Interestingly, it is worth noting that this pattern did not hold true for men. In other words, men’s perceptions of emotional invalidation did not have a similar impact on their partner’s relationship satisfaction. This gender difference in the direct partner effect of emotional invalidation may be due to various factors. It is possible to hypothesize that because men tend to rely on withdrawal and avoidance to deal with stress, while women tend to engage and talk about it with their partners (Schulz et al., 2004), the experience of invalidation may have less impact on women’s relationship satisfaction. However, further research is needed to explore why this gender-specific pattern emerged.

Our H5, which anticipated a relationship between one’s own perceived emotional invalidation and their partner’s psychological distress, was not supported. While a previous study suggested that emotion processes are likely to influence own and partner symptoms of adjustment disorder and depression (Horn & Maercker, 2016), we did not find this pattern of result. Specifically, while we observed actor effects, indicating that an individual’s own emotional experiences influenced their own psychological distress, we did not find significant partner effects. This discrepancy suggests that emotional processes may impact individual adjustment differently than they do partners’ psychological distress. Specifically, our assessment of perceived emotional invalidation focused on a very personal experience of feeling invalidated or criticized, which appears to affect an individual’s own psychological distress more than it influences their partner’s psychological distress.

Regarding the hypothesis concerning the influence of one’s own psychological distress on their partner’s relationship satisfaction (H6), our findings partially confirmed this hypothesis, indicating a significant effect only among women. While previous studies have found this type of partner effect but without gender differences (Gana et al., 2016; Whisman et al., 2004), in our study the partner effect occurred only from women’s psychological distress to men’s relationship satisfaction. One possible explanation is that women’s psychological distress or difficulties related to distress may increase their need for affiliation or social support (Duncan & Peterson, 2010), potentially increasing men’s burden and thereby decreasing men’s relationship satisfaction.

Regarding actor indirect effects, our H7 was partially confirmed. Specifically, among women, the perception of emotional invalidation was found to be linked to their own relationship satisfaction through the pathway of their own psychological distress, aligning with our expectations (Bodenmann et al., 2007; Falconier et al., 2015; Ledermann et al., 2010). This phenomenon can be attributed to the intricate interplay between emotional invalidation, psychological distress, and couple relationship satisfaction, which may manifest differently in women compared to men. It suggests that for women, emotional invalidation might trigger psychological distress (Brandão et al., 2022; Schreiber & Veilleux, 2022; Zielinski et al., 2022), which in turn impacts their overall relationship satisfaction (Bodenmann et al., 2007; Tolpin et al., 2006). For men, the mediational hypothesis was not confirmed. Although perceived emotional invalidation in men was associated with their individual psychological distress, this distress did not show a significant association with their relationship satisfaction. The differing impact of psychological distress on relationship satisfaction for men and women can be attributed to several factors. Women generally tend to express their emotions more openly than men (Chaplin, 2015), which can make their psychological distress more evident within the relationship. This openness may affect their interactions with their partner, thereby impacting their overall relationship satisfaction. In contrast, men tend to internalize their distress, which can result in a disconnect between their internal emotional state and their outward behaviour, allowing them to maintain relationship satisfaction despite experiencing distress.

It is also possible to hypothesize that men may benefit from the support provided by women to deal with the psychological distress with less repercussion on their relationship satisfaction, as previous studies suggest that women often display a greater capacity for regulating other people’s emotional distress compared to men (e.g., Bodenmann et al., 2015). Supportive partners can play a crucial role in helping individuals cope with emotional challenges. When partners are responsive, understanding, and provide a safe space for emotional expression, it can mitigate the negative impact of distress on relationship satisfaction, including the distress related to feeling misunderstood or invalidated by others. Also, it is possible that other variables not accounted for in the study, such as individual coping strategies or relationship dynamics, played a moderating role in this relationship.

Finally, our H8 was also partially confirmed since own perceived emotional invalidation was associated with partners’ relationship satisfaction via own psychological distress but also only for women. This suggests that when women feel emotionally invalidated within the relationship, it can lead to higher psychological distress, which subsequently affects the men’s overall satisfaction with the relationship. These findings align with the spillover and contagion effects theories (Bodenmann, 1995; Randall & Bodenmann, 2017; Schoebi & Randall, 2015). These results are consistent with those found by Falconier et al. (2015), where individuals’ daily stressors increased their own intraindividual distress, which in turn influenced their partner’s relationship satisfaction. This effect was particularly pronounced for women, suggesting that women’s stressors and distress pose greater risks to the couple’s relationship than men’s stressors and distress. Overall, it seems that the emotional distress experienced by women due to perceived emotional invalidation may spill over into the relationship and influence their male partners’ satisfaction. This could occur because emotional distress can impact the overall emotional climate of the relationship, making it less satisfying for both partners. Previous studies (e.g., Duncan & Peterson, 2010; Neff & Karney, 2005; Schulz et al., 2004) have found that women tend to engage more in communication and seek more support when experiencing distress, which can contribute to decrease their partners’ relationship satisfaction.

In summary, our findings underscore the significant impact of perceived emotional invalidation on individuals’ psychological distress. Moreover, our study reveals that these effects can manifest differently in terms of relationship satisfaction, particularly with women’s psychological distress potentially influencing men’s relationship satisfaction. Despite the valuable insights provided by the dyadic approach in this study, it is essential to acknowledge several limitations that may impact the interpretation of the results. Firstly, the cross-sectional design employed in this research precludes us from establishing causal relationships among the study variables. While the dyadic approach provides a comprehensive understanding of the dynamics within couples, it is crucial to consider the temporal sequence of perceived emotional invalidation, psychological distress, and relationship satisfaction to draw more definitive conclusions about causality. Additionally, relying solely on self-report measures may introduce biases in how individuals report their perceptions of perceived emotional invalidation, psychological distress, and relationship satisfaction, potentially influencing the results. Potential alternatives to self-report measures include observational methods, in which trained researchers code interactions between partners for indicators of relationship quality and emotional invalidation. Additionally, physiological measures, such as cortisol levels or heart rate variability, can provide objective data on stress and emotional responses. Future dyadic studies could benefit from a multimethod approach that combines self-report, observational, and physiological data to capture a more comprehensive picture of relationship dynamics.

Additionally, using a single item to measure relationship satisfaction is a limitation. A single-item measure may not capture the complexity and multidimensionality of relationship satisfaction. Future research should employ validated multi-item scales that assess various aspects of relationship satisfaction, such as the Dyadic Adjustment Scale (DAS) or the Relationship Assessment Scale (RAS). These scales provide a more reliable and nuanced understanding of relationship satisfaction, enhancing the robustness of the findings.

Furthermore, it is worth noting that the PIES assesses perceived emotional invalidation in a broader context, encompassing responses from various individuals, not only romantic partners. While this study did not explicitly specify that participants should respond in the context of their partners, it is conceivable that their perceptions of emotional invalidation by others may also include their perceptions of emotional (in)validation within their romantic relationships. Future research should aim to disentangle these associations to gain a more nuanced understanding of these dynamics.

Secondly, the study delves into the exploration of one psychosocial process that might mediate the relationship between perceived emotional invalidation and relationship satisfaction – psychological distress. However, it appears that other important psychological processes may also be involved in these associations, such as support provision or communication patterns. Also, it is plausible that specific emotion regulation strategies, as suggested in prior research (e.g., Brandão et al., 2022), could elucidate these associations. Further investigations are warranted to explore these potential mechanisms both at the individual level and within the context of couples, deepening our understanding of the intricate pathways through which perceived emotional invalidation affects psychological distress and, consequently, couple relationship satisfaction.

Lastly, it is worth noting that the levels of perceived emotional invalidation and psychological distress observed in both men and women were relatively low in this sample, while levels of relationship satisfaction were relatively high. Therefore, caution should be exercised when interpreting the results, as the pattern of associations may differ with higher levels of psychological distress. Additionally, couples who agreed to participate in this study may have higher levels of relationship satisfaction. Future studies with diverse and more representative samples are needed to confirm and extend the findings observed in this research.

This study’s clinical implications are particularly relevant for therapists and counsellors working with couples facing challenges related to emotional dynamics and perceived emotional invalidation. Firstly, it underscores the importance of recognizing and addressing perceived emotional invalidation as a potential source of distress within relationships. Therapists should proactively promote open and empathetic communication between partners. Our findings indicate that women’s relationship satisfaction is directly and indirectly influenced via psychological distress by perceived emotional invalidation. Therefore, helping couples identify sources and patterns of emotional invalidation can reduce women’s psychological distress and eventually improve couples’ relationship satisfaction for both members of the couple (considering the indirect partner effect found).

Moreover, the gender-specific effects revealed in this study emphasize the need for tailored interventions. When working with couples, therapists should consider that men may benefit from improved emotional support and guidance in understanding and responding to their partner’s distress. Additionally, women might benefit from interventions that help them effectively manage their own psychological distress.

## Declaration of generative AI and AI-assisted technologies in the writing process

During the preparation of this work the author(s) used ChatGPT to improve readability and language. After using this tool, the author(s) reviewed and edited the content as needed and take(s) full responsibility for the content of the publication.

## Data Availability

Data presented in this study is available on request from the corresponding author. The data is not publicly available due to privacy and ethical restrictions.*
